# Amperometric Biosensor for Oxalate Determination in Urine Using Sequential Injection Analysis

**DOI:** 10.3390/molecules17088859

**Published:** 2012-07-26

**Authors:** Jose A. Rodriguez, Prisciliano Hernandez, Veronica Salazar, Yolanda Castrillejo, Enrique Barrado

**Affiliations:** 1 Chemical Research Center, Universidad Autonoma el Estado de Hidalgo, Carr. Pachuca-Tulancingo km 4.5, 42076, Pachuca, Hidalgo, Mexico; Email: pris656_1@hotmail.com (P.H.); salazar@uaeh.edu.mx (V.S.); 2 Analytical Chemistry Department, Faculty of Sciences, Universidad de Valladolid, Campus Miguel Delibes s/n, 47011 Valladolid, Spain; Email: ycastril@qa.uva.es (Y.C.); ebarrado@qa.uva.es (E.B.)

**Keywords:** oxalate, urine, sequential injection analysis, magnetic particles

## Abstract

An amperometric flow biosensor for oxalate determination in urine samples after enzymatic reaction with oxalate oxidase immobilized on a modified magnetic solid is described. The solid was magnetically retained on the electrode surface of an electrode modified with Fe (III)-tris-(2-thiopyridone) borate placed into a sequential injection system preceding the amperometric detector. The variables involved in the system such as flow rate, aspired volumes (modified magnetic suspension and sample) and reaction coil length were evaluated using a Taguchi parameter design. Under optimal conditions, the calibration curve of oxalate was linear between 3.0–50.0 mg·L^−1^, with a limit of detection of 1.0 mg·L^−1^. The repeatability for a 30.0 mg·L^−1^ oxalate solution was 0.7%. The method was validated by comparing the obtained results to those provided by the spectrophotometric method; no significant differences were observed.

## 1. Introduction

Measurement of oxalate in urine is of clinical interest, as an increase in oxalate excretion through urine indicates hyperoxaluria, renal failure, kidney lesions and pancreatic insufficiency [1]. Different analytical techniques have been applied for quantification of oxalate in urine such as enzymatic spectrophotometric, liquid and gas chromatography, capillary electrophoresis and ion chromatography [2,3,4,5,6,7]. Enzyme based biosensors have attracted attention in different areas due to good selectivity, sensitivity and reliability [3]. Oxalate biosensors are usually based on the analysis of reaction products generated by reaction between oxalate and oxalate oxidase (OxOx) in presence of oxygen, according to the following reaction:


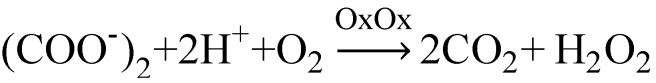
(1)


According to equation (1), oxalate concentration can be determined indirectly by measuring the change in pH (proportional to CO_2_ concentration), or by measuring the hydrogen peroxide concentration spectrophotometrically [8] or electrochemically [9]. 

Biosensors using direct determination of enzymatically produced hydrogen peroxide have been avoided in clinical samples because they require high potentials during detection (>1.0 V) causing interference produced by ascorbic acid, uric acid and other components of the sample. This disadvantage has been overcome by using electron-mediated enzyme electrodes. The mediators more used in biosensors include ferricyanides, phtalocyanines and ferrocenes [10]. Potassium hydrotris (2-thiopyridone) borate (KTpm) is a flexible ligant similar to cyclopentadienyl due to the same charge and number of electrons donated (anionic, 6e^−^ donor) as well as the facial geometry typically adopted [11,12]. The use as mediators of metal complexes based on this ligant has not been described.

Oxalate enzyme-derived biosensors based on mediator modified electrodes were fabricated and studied. However, the fabrication process is complex and the reproducibility is poor. Moreover, the regeneration of the electrode is slow, especially in flowing systems such as flow injection and liquid chromatography. The concept of sensors based on injection of the recognition element (in this case OxOx), using an internal chamber with enzyme in solution, has been applied to solve the regeneration problem, but the enzyme consumption is higher than using it immobilized [13,14]. This concept might be exploited mainly using sequential injection analysis (SIA) [15]. This flow technique has advantaged over flow injection systems in reducing reagent consumption because SIA is based on non-continuous flow [16]. The syringe pump in SIA allows the use of small volume in a sequential mode.

The development and application of magnetic solid in separation and detection methodologies has attracted interest in the last years. This is mainly due to the versatility, high surface area, chemical and physical stability and low toxicity [17,18]. The interaction of adequate external magnetic field with suspended magnetic solids flowing through the system, allows retention of the solid phase into a specific area promoting their analytical application [19]. 

In this work, we propose a sequential injection biosensor with amperometric detection for oxalate determination in urine based on an electrode modified with Fe (III)-tris(2-thiopyridone)borate complex as mediator coupled with injection of magnetic solid modified with OxOx, as recognition element, transiently retained on the electrode surface subjected to an external magnetic field. The developed system was tested in human urine samples and showed to be adequate concerning robustness, sampling rate and quality of analytical results. 

## 2. Results and Discussion

### 2.1. Electrocatalytic Properties of Fe-Tmp Modified Electrode for H_2_O_2_ Detection

**Figure 1:**
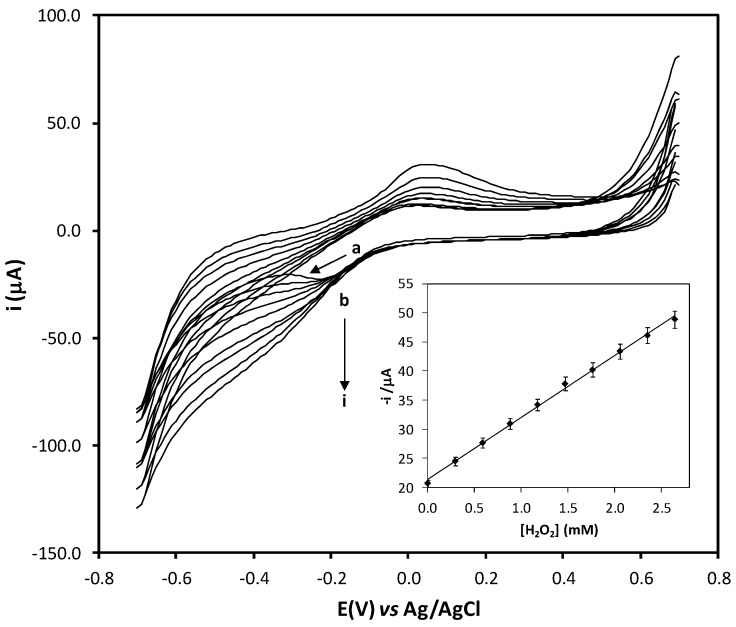
Cyclic voltammograms of Fe-Tmp modified electrode in acetate buffer solution (pH 4.0. 0.1M) at scan rate 50 mV·s^−1^ for different H_2_O_2_ concentrations from 0.0 to 2.6 mM. Inset: plot of cathodic peak current (µA) *vs.* [H_2_O_2_] (mM).

Figure 1a shows cyclic voltammograms of a Fe-Tmp modified electrode in acetate buffer solution (pH 4.0, 0.1 M) in absence and presence of hydrogen peroxide. A defined redox couple (E^o^’ = −0.09 V), corresponding to Fe (III)’/Fe (II)’ system can be observed. As shown (Figure 1b–i) an increment in the concentration of H_2_O_2_ generates an increment in the cathodic peak height while in the anodic peak a decrease is observed. The catalytic currents increased linearly with the H_2_O_2_ concentration of the range of 0.1 to 2.6 mM using cyclic voltammetry. Lower concentration ranges can be achieved using amperometric detection. The reversibility, stability, reproducibility and fast electron transfer kinetics of the Fe-Tmp modified electrode are appropriate for its use as mediator during detection of hydrogen peroxide at low concentration levels at applied potential of −0.20 V. 

The effect of pH on the electrocatalytic response was studied using Britton-Robinson buffer solution (0.1 M) in the concentration range from 2.0 to 10.0 containing 1.0 mM of H_2_O_2_. The cathodic current increased with increasing pH up to 4.0, and then remained constant between 4.0 and 5.0. At higher pH values a decrease in the cathodic peak is observed likely due to the iron hydrated oxide formation. Therefore, an optimal pH value of 4.0 (acetate buffer solution) was chosen. This pH value corresponds to the value used during enzymatic determination of oxalate using OxOx [9]. The influence of the applied potential to the working electrode on the catalytic response was evaluated using amperometric detection. The amperometric signal increased using applied potentials from 0.0 to −0.2 V and then remained constant at lower values (−0.3 to −0.5 V). An applied potential of −0.2 V was therefore selected in an effort to minimize the interference produced using lower detection potential.

### 2.2. Optimization of the System Variables

**Figure 2:**
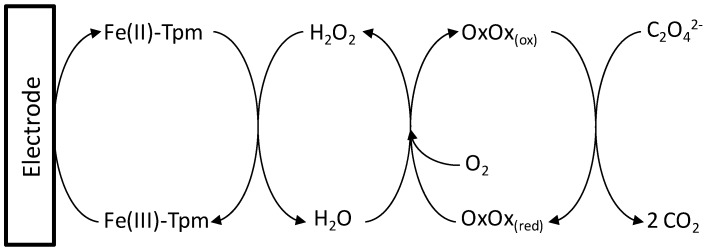
Reaction scheme for redox mediated oxalate determination.

Oxalate is determined according the following sequence of reactions (Figure 2). Several factors affecting the proposed system required optimization [20,21]. A Taguchi parameter design (TPD) was selected as the robust optimization method since this provides the necessary information with the minimal experimentation. This design discriminates between control factors and experimental noise through orthogonal arrays in which the columns (factors) and rows (trials) are arranged in a conveniently fixed manner. These matrices allow the simultaneous evaluation of several variables with the minimum number of trials. The results obtained were analyzed statistically to obtain the optimum of each variable. 

Optimization of the system with TPD involves five steps, (a) identifying the output variable to optimize; (b) identifying and selecting factors that affect the system; (c) selecting the appropriate orthogonal array and assigning adequate settings to the chosen variables; (d) analyzing the data and determining the optimum settings and (e) conducting a confirmatory experiment under the optimal conditions obtained.

In analytical flow techniques with amperometric detection, the desired response is the maximum signal height (current, µA). The flow variables optimized in sequential injection systems are the enzyme concentration (aspirated volume of enzyme magnetic suspension), the flow rate of the carrier solution, the aspirated sample volume and the reactor length from the selection valve to the detector. The selected orthogonal array must have a number of columns equal to or higher than the number of degrees of freedom of the system; thus an L_9_(3^4^) array was used. The three settings selected for each factor were chosen taking into account preliminary analyzes. 

The flow rate (Q) has been shown in enzymatic flow systems that control the H_2_O_2_ production during the enzymatic reaction. SIA with enzymatic bead injection requires the use of flow rates <1.0 mL·min^−1^ in order to increase the H_2_O_2_ generation [10]. The reactor length (RL) must be sufficiently long for conditioning the sample pH. With respect to the aspired volumes for sample (SV) and enzyme magnetic suspension (MV), they are critical in flow systems; therefore, those selected must be large enough to allow the correct dispersion of the sample with the carrier solution and OxOx concentration on the modified electrode.

Table 1 shows factorial design matrix, the settings for each experiments used for the optimization experiments and the results of the peak height for each trial. All experiments were performed in triplicate in order to calculate the residual error; the total number of experiments was therefore 27 (9 experiments × 3 replicates). Measurements were performed with solutions containing 50.0 mg·L^−1^ of oxalic acid.

**Table 1:** L_9_(3^4^) orthogonal array and peak height in each experiment.

MV (µL) ^a^	Q (mL·min^−1^)	RL (cm)	SV (µL)	Peak height, µA (%RSD, n = 3)
10	0.50	30	100	1.54 (0.23)
10	0.75	50	150	2.13 (0.05)
10	1.00	70	200	2.62 (0.10)
20	0.50	50	200	1.77 (0.08)
20	0.75	70	100	0.71 (0.28)
20	1.00	30	150	1.53 (0.26)
30	0.50	70	150	0.38 (0.37)
30	0.75	30	200	0.59 (0.09)
30	1.00	50	100	0.40 (0.13)

^a^ [OxOx] = 0.7 U·mL^−1^.

The results were analyzed statistically to adjust each variable to its optimum with least variable possible. All calculations were made using ANOVA-TM v2.5 software. Table 2 shows the results for this analysis. The values of the variance ratio (*F*) and the critical variance ratio (4.56, α = 0.05) show that all the factors taken into account were critical (F_calculated_ > F_critical_). The factor with a greatest influence on the response was the enzyme magnetic suspension volume, which contributes 77.34% of the total variance, followed by the sample volume (16.82%). The contribution of the residual error was 0.01%, this indicates the correct selection of experimental parameters.

**Table 2:** Analysis of variance for peak height in Table 1.

Variance source	Variance	Variance ratio ( *F*) ^a^	Influence (%) ^b^
MV	6.300	11340	77.34
Q	0.345	621	4.24
RL	0.130	234	1.60
VI	1.370	2466	16.82
Residual	0.001		0.01

^a^ The critical variance ratio for a 95% confidence level is 4.56 (2,18); ^b^ Influence is defined as 100× (variance/total sum of variances).

**Figure 3:**
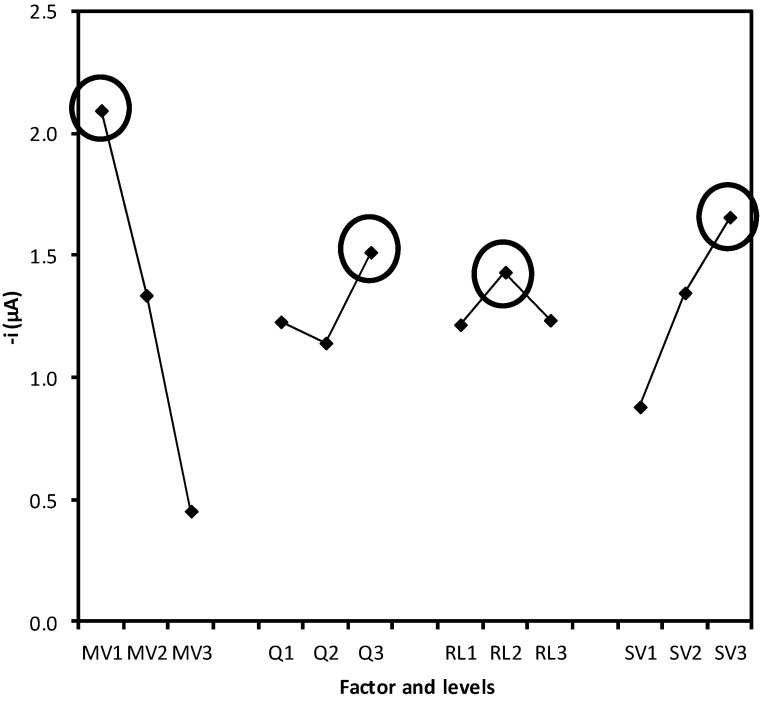
Effects of control factors on the mean peak height (µA) signal. MV, enzyme magnetic suspension volume; Q, flow rate; RL, reaction length; SV, sample volume.

Figure 3 shows the effects of the control factors on the output variable, among which the aspired volumes are the most important (longer lines in comparison with the other variable). The combination of settings that allowed the highest output variable (highest response in Figure 3) was, MV, 10 µL; Q, 1.00 mL·min^−1^; RL, 50 cm; SV, 200 µL. The flow rate used to aspirate the solutions (30 mL·min^−1^) helps to promote a turbulent flow, which contributes the mixing of the sample and the carrier solution, increasing the sampling rate of the method. 

### 2.3. Analytical Properties of the Procedure

**Figure 4:**
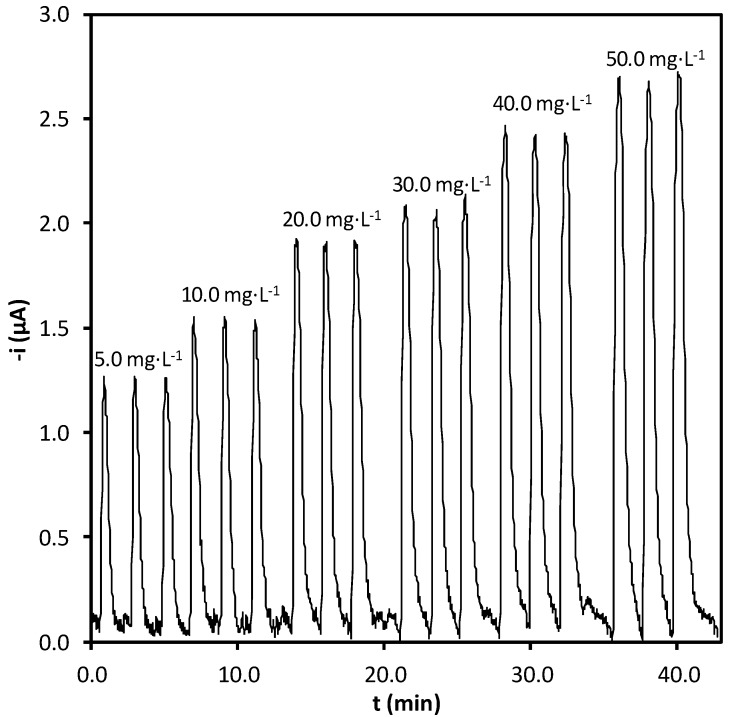
Recorder output obtained with the proposed system, providing an oxalate calibration plot between 5.0 and 50.0 mg·L^−1^.

Using the SIAgram obtained under optimal conditions (Figure 4), a standard curve for oxalate was constructed using the mean peak height (µA) values of each solution. Table 3 shows the regression parameters taken from this standard curve. The limit of detection was calculated according to IUPAC criteria [22] *i.e.*, 3.29S_e_/b1, where S_e_ is the square root of the residual variance of the standard curve, and b_1_ is the slope. The intermediate precision of the procedure, expressed as the relative standard deviation (%RSD), for three determinations (made on different days) using synthetic samples with analyte concentration of 30 mg·L^−1^ was 2.2%. The SIAgram was used to calculate the repeatability of the determinations; the RSD was below 2% for all standard solutions and samples-under optimum conditions 30 determinations per hour can be analyzed. The modified electrode was used for a week and the enzyme magnetic solid was renewed daily.

The interference possible compounds present in the urine samples such as: urea, uric acid, ascorbic acid and salicylic acid was studied. Solutions containing 20.0 mg·L^−1^ of oxalate and the foreign compound at higher concentrations (maximum 100:1) were analyzed. The interference concentration of each compound was considered as the one which caused a variation in the response greater than or equal to ±5% compared to the response obtained in its absence. The results demonstrate that none of these compounds interfered in the determination. The limit of detection and the linear range confirmed that SIA-biosensor provides adequate sensitivity to determine oxalate at the normal interval (0.0–20.0 mg·L^−1^ in healthy patients and >20.0 mg·L^−1^ for stone formers) of concentration in urine samples [1,23].

**Table 3:** Regression values for the calibration plot, peak height (µA) *vs.* [oxalate] (mg·L^−1^).

Parameter	Value
Square root of residual variance, S_e_	0.009
Determination coefficient, r^2^	0.999
Intercept confidence interval, b_0_ ± ts (b_0_)	0.017 ± 0.019
Slope confidence interval, b_1_ ± ts(b_1_) (µA L·mg^−1^)	0.031 ± 0.001
Linear range (mg·L^−1^)	3.0–50.0
Limit of detection (mg·L^−1^)	1.0
Repeatability (%RSD, n = 3, 30 mg·L^−1^)	0.7
Intermediate precision (%RSD, n = 3, 30 mg·L^−1^)	2.2
Sampling rate (samples·h^−1^)	30

The proposed method was used to determine oxalate in urine samples from apparently healthy male and female in three age groups: children (1−20 year), young (21−45 year) and old (>45 year), (n = 3 in each group). The results were compared to the flow injection analysis with spectrophotometric detection (FIA-Vis). For each sample, the mean oxalate concentrations (n = 3) obtained with the two methods were compared using the Student t test, assuming comparable variances (confirmed by an F test). The values of t_experimental_ were then compared to a t_tabulated_ at 95% confidence interval (t = 2.78, α = 0.05, n = 4). No significant differences were seen between the results provided by each method. Results show that urinary oxalate values were in range 9.2–42.8 mg·L^−1^, in healthy male and female, respectively. These values are comparable to those reported in earlier studies [24]. 

**Table 4:** Concentration of urinary oxalate (mg·L^−1^, mean ± confidence interval) in healthy individuals determined by SIA and spectrophotometric method.

Age group (years)	Sex	[Oxalate] (mg·L^−1^)		t_experimental_
		SIA	FIA-Vis	
Children (1−20)	Male	10.5 ± 4.0	10.8 ± 4.3	0.15
	Female	17.2 ± 3.8	17.4 ± 1.9	0.19
Adult (21−45)	Male	21.0 ± 6.3	20.5 ± 2.2	1.35
	Female	20.3 ± 1.2	21.1 ± 2.3	1.23
Old (>45)	Male	23.5 ± 2.3	23.6 ± 2.4	0.08
	Female	37.4 ± 4.1	36.8 ± 3.5	0.15

t_tabulated_ = 2.78.

## 3. Experimental

All solutions were prepared by dissolving the corresponding analytical grade reagent in deionized water (<100 µS·cm^−1^) and were used without further purification. A stock solution (1.0 g·L^−1^) of oxalic acid was prepared weekly by dissolving the salt in water. Standard solutions ranging from 5.0 to 50.0 mg·L^−1^ were prepared daily by dilution of this stock solution. Acetate buffer solution (pH 4.0, 0.1 M) was used as carrier solution. Oxalate oxidase (0.7 units/mg solid), glutaraldehyde (mass fraction 25%), 3-aminopropylammonium bromide, Triton X-100, tetramethylorthosilicate, chloroform, hexane were obtained from Sigma Aldrich (St. Louis, MO, USA). FeSO_4_·7H_2_O, FeCl_3_·6H_2_O, NaOH, methanol, CaCl_2_·2H_2_O, and NH_3_ were of analytical grade (Merck, Xalostoc, Mexico). The K^+^ [hydrotris (2-thiopyridone) borate]^−^ was obtained according to Dyson *et al.* [11].

### 3.1. Synthesis of Enzyme Magnetic Solid

Magnetite (0.80 g) synthesized by co-precipitation [25] is added to a reactor containing tetramethoxysilane (0.25 g) and 3-aminopropylmethoxysilane (1.25 g). The monomers were dissolved in 24 mL of a solution containing 2% (w/v) Triton X-100, 12.5% (v/v) methanol, 0.02% (w/v) cetyltrimethylammonium bromide and 200 µL NH_3_ (28% w/v) as catalyst. The mixture was heated and refluxed for 16 h with stirring. The NH_2_ modified magnetic solid was washed with 10.0 mL of each of the following solvents: ethanol and water. The obtained solid was dried at 60 °C for 24 h. 

The OxOx was immobilized on the magnetic support as follows: the NH_2_ modified magnetic solid (50 mg) was mixed with glutaraldehyde solution (2.0%, 5 mL). The mixture was stirred and after 2 h, the solid was washed with deionized water (previously deoxygenated, 20 mL) followed by phosphate buffer solution (pH 7.0, 0.1 M, 20 mL), also previously deoxygenated. The support was suspended in phosphate buffer solution (5.0 mL) containing OxOx (3.5 U, 500 µL) and the dispersion was stirred for 6 h. The magnetic suspension obtained was used in the sequential injection system. 

### 3.2. Characterization of the Magnetic Solid

**Figure 5:**
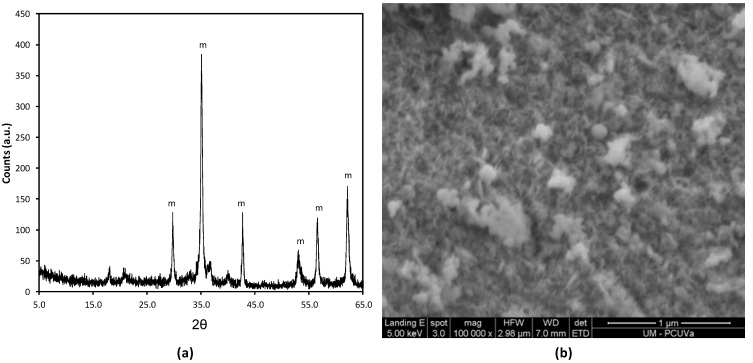
(**a**) Diffraction pattern and (**b**) Scanning electron microscopy image for the magnetic solid.

Figure 5 shows the diffraction pattern (obtained in a Phillips PW1710 diffractometer, Almelo, Holland) for the magnetic solid shows reflections corresponding to magnetite (“m” in Figure 1). In addition, the presence of a wide band at 2θ angles from 15° to 25° is consistent with the amorphous phase of the silica [17]. The overall morphology of the magnetic solid was studied by SEM (Jeol JSM-820, Peabody, MA, USA). The solid has a spherical morphology (Figure 5b). The size distribution of the particles (dried at 100 °C for 24 h) was determined to be in the range of 50 to 150 nm based on the measurements of the sizes of particles. 

### 3.3. Synthesis of Fe (III)-Tpm

A methanolic solution of FeCl_3_·6H_2_O (25 mg, 0.09 mmol, 5 mL) was added to a methanolic solution of K^+^[hydrotris(2-thiopyridone)borate]^−^ (68 mg, 0.09 mmol, 5 mL). A yellow brown precipitate was formed immediately [11]. The suspension was stirred for 12 h, the precipitate was extracted twice with chloroform (5 mL) and the organic phase was evaporated at room temperature. The resulting solid was washed with hexane (5 × 4 mL) [26]. Yield: 59 mg (0.07 mmol, 78%). Infrared characteristic vibrations: nB-H = 2,510; nC-H_3_ = 2,850; nC-H_th_ = 2,920 cm^−1^. The NMR studies suggested a paramagnetic nature for this complex.

### 3.4. Apparatus

**Figure 6:**
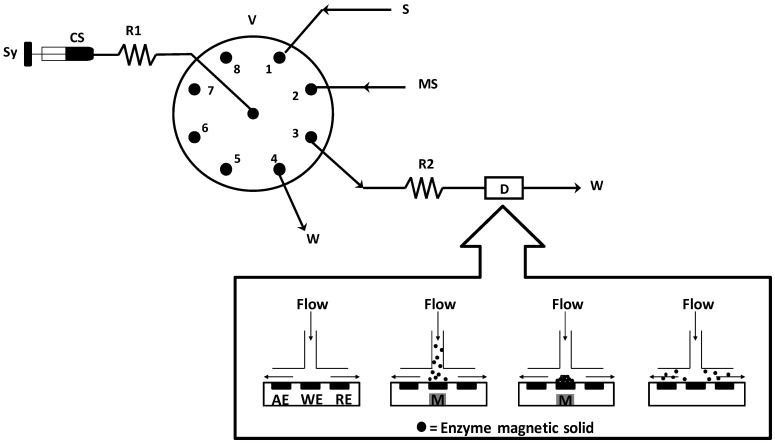
Diagram of the SIA system used to determine oxalate. Sy, syringe; CS, carrier solution; R1, loading reactor; V, selection valve; S, sample; MS, enzyme magnetic suspension; R2, reaction coil; D, detector; W, waste; AE, auxiliary electrode, WE, working electrode; RE, reference electrode; M, magnet.

The sequential injection system (Figure 6) consists of a burette multisyringe with programmable speed (S, microBu 2030, Crison, Barcelona, Spain) used to aspire and dispense the reagent solutions, an eight-way selection valve (V, Crison) and a bipontentiostat (D, Drop Sens μSTAT 200) as detector. The different components of the flow manifolds were connected using Omnifit Teflon tubing (0.8 mm i.d.) and Gilson connectors. The instrumental devises were controlled by Autoanalysis 5.0 software. 

The analytical cycle began with the aspiration of enzyme magnetic suspension (MS, 10 µL) onto the loading reactor (R1, 100 cm) at a flow rate of 30 mL·min^–1^. This was then directed towards the detector, subjected to an external magnetic field, by the carrier solution (CS). These steps were repeated to renew the recognition agent on the electrode surface. A neodymium magnet (Nd-Fe-B, 3.0 mm × 4.0 mm d.e., 1.3 T) was placed at the bottom of the working electrode. The magnet was removed during renew of the recognition agent. 

Once the biosensor has been formed, sample solution (200 µL) was aspired. The sample solution (S) was mixed with the carrier solution in the reactor 2 (R2, 50 cm) and detected at −0.2 V using the modified electrode at 1.00 mL·min^−1^. The screen printed modified carbon electrode (℘ = 4 mm) was performed by impregnating 20 μg of Fe(III)-Tpm (in acetonitrile) on the surface of working electrode (Drop Sens 110A) and drying overnight at room temperature. A wall-jet electrochemical cell (Drop Sens DRP-FLWCL) was used as detector.

The concentration of oxalate in the analyzed samples was also determined for comparative purposes using flow injection analysis with spectrophotometric detection (FIA-Vis), this method was previously validated to analyze oxalate in urine samples. The FIA system consists of a four-channel Gilson Minipuls 2 peristaltic pump fitted with propulsion tubes of the same brand used to propel sample and carrier solutions. A sample volume of 250 µL was injected into the Fe (III) solution (1.0 × 10^–3^ M, in H_2_SO_4_ 0.01 M) using a Rheodyne 5020 four-way rotary valve. The reduction of the Fe (III)-oxalate complex takes place in the photochemical reactor (250 cm × 0.8 mm i.d.). The Fe (II) produced reacts with the ferrozine solution (1 × 10^−3^ M, in acetate buffer solution pH 4.0, 0.2 M) in a reaction coil (25 cm × 0.8 mm i.d.). The Fe (II)-ferrozine complex was detected at 562 nm is an UV-visible spectrophotometer (Lambda 40, Perkin-Elmer, Norwalk, CT, USA) with a Hellma 178.712QS 18 µL flowthrough cell. The flow rate of each individual channel was 0.6 mL·min^−1^ [27].

Urine samples (5.0 mL) were mixed with saturated CaCl_2_ solution (2.0 mL). The mixture was centrifuged 5 min at 2,500 rpm. The liquid phase was discarded and the precipitate was dissolved in H_2_SO_4_ 0.1 M (1.0 mL). The solution obtained was injected into the FIA-Vis manifold for its analysis. Oxalate concentration was determined by interpolation using an oxalate standard curve constructed in the interval from 10.0 to 50.0 mg·L^−1^.

## 4. Conclusions

The results presented in this paper indicate that Fe (III)-Tpm modified electrodes can be used for H_2_O_2_ determination. The automatic system developed allows the preparation of renewable oxalate biosensor on-line by immobilizing enzyme magnetic solids on the modified electrode surface to form a sensitive surface. Small volumes of reagents (10 µL) and sample (200 µL) are used. The method is simple, robust, economic, and does not require sample treatment. The results obtained show no differences from reference procedures. Hence, the presented approach is an advantageous alternative to the batch analysis. It may be anticipated that other magnetic biomaterials may also be synthesized for fabrication of other useful biosensors. 
